# A prediction model for renal artery stenosis using carotid ultrasonography measurements in patients undergoing coronary angiography

**DOI:** 10.1186/1471-2369-15-60

**Published:** 2014-04-14

**Authors:** Yonggu Lee, Jeong-Hun Shin, Hwan-Cheol Park, Soon Gil Kim, Seong-il Choi

**Affiliations:** 1 Department of Cardiology, Hanyang University Guri Hospital, Guri City, Kyeungg-do, Republic of Korea

**Keywords:** Renal artery stenosis, Coronary artery disease, Carotid atherosclerotic plaque, Carotid intima-media thickness, Prediction model

## Abstract

**Background:**

Carotid intima-media thickness (CIMT) and carotid atherosclerotic plaque (CAP) are well-known indicators of atherosclerosis. However, few studies have reported the value of CIMT and CAP for predicting renal artery stenosis (RAS). We investigated the predictive value of CIMT and CAP for RAS and propose a model for predicting significant RAS in patients undergoing coronary angiography (CAG).

**Methods:**

Consecutive patients who underwent renal angiography at the time of CAG in a single center in 2011 were included. RAS ≥50% was considered significant. Multiple logistic regression analysis with step-down variable selection method was used to select the best model for predicting significant RAS and bootstrap resampling was used to validate the best model. A scoring system for predicting significant RAS was developed by adding the closest integers proportional to the coefficients of the regression formula.

**Results:**

Significant RAS was observed in 60 of 641 patients (9.6%) who underwent CAG. Hypertension, diabetes, significant coronary artery disease (CAD) and chronic kidney disease (CKD) stage ≥3 were more prevalent in patients with significant RAS. Mean age, CIMT and number of anti-hypertensive medications (AHM) were higher and body mass index (BMI) and total cholesterol level were lower in patients with significant RAS. Multiple logistic regression analysis identified significant CAD (odds ratio (OR) 5.6), unilateral CAP (OR 2.6), bilateral CAP (OR 4.9), CKD stage ≥3 (OR 4.8), four or more AHM (OR 4.8), CIMT (OR 2.3), age ≥67 years (OR 2.3) and BMI <22 kg/m^2^ (OR 2.4) as independent predictors of significant RAS. The scoring system for predicting significant RAS, which included these predictors, had a sensitivity of 83.3% and specificity of 81.6%. The predicted frequency of the scoring system agreed well with the observed frequency of significant RAS (coefficient of determination *r*^2^ = 0.957).

**Conclusions:**

CIMT and CAP are independent predictors of significant RAS. The proposed scoring system, which includes CIMT and CAP, may be useful for predicting significant RAS in patients undergoing CAG.

## Background

Renal artery stenosis (RAS) increases the risk of mortality in patients with cardiovascular disease. RAS is associated with the prevalence and severity of coronary artery disease (CAD) [1-3], and is a correctable cause of severe hypertension and ischemic nephropathy [4]. However, RAS remains under-recognized, because most patients with RAS have no symptoms or signs. RAS is more prevalent in patients undergoing coronary angiography (CAG) than in the general population [5]. Performing renal angiography at the time of CAG can be a safe, cost-effective diagnostic strategy in patients at high risk of significant RAS [6]. However, routine evaluation for RAS in asymptomatic patients undergoing CAG is difficult to justify, because of the lack of evidence for clinical benefits associated with renal artery intervention in patients with RAS. An advisory from American Heart Association for renal angiography at the time of CAG focuses on occasional cases with symptoms or clues suggesting RAS [6]. The indications for investigation for RAS at the time of CAG in asymptomatic patients have not been established.

Carotid intima-media thickness (CIMT) and carotid atherosclerotic plaque (CAP) are well-known indicators of systemic atherosclerosis [7,8]. Although several studies have proposed models for predicting significant RAS using clinical parameters such as CAD, age, peripheral artery disease (PAD), and kidney function in patients undergoing CAG [9-11], no studies have reported the value of ultrasonography measurements of CIMT or CAP for predicting RAS. The aims of this study were to determine whether CIMT and CAP can predict RAS, and to propose a prediction model for RAS using these carotid ultrasonography measurements in patients undergoing CAG.

## Methods

### Study subjects and baseline data collection

From January to December 2011, consecutive patients undergoing elective CAG at Hanyang University Guri Hospital were prospectively included in this study. Patients with end-stage renal disease, patients undergoing emergency percutaneous coronary intervention (PCI), and patients with a history of renal artery intervention were excluded. Written informed consent was obtained from all patients at the time of enrollment in the study. All patients underwent simultaneous coronary and renal angiography. They also completed physical examinations including measurement of blood pressure, body weight and height, and laboratory tests including serum creatinine level, lipid profiles, hemoglobin A1c level and urinalysis. Body mass index (BMI) was calculated as (weight)/(height)^2^ (kg/m^2^). Proteinuria was defined as a random urine protein/creatinine ratio of >300 mg/g. The estimated glomerular filtration rate (eGFR) was calculated using the Modification of Diet in Renal Diseases equation. The classification of chronic kidney disease (CKD) stages stated in Kidney Disease Outcomes Quality Initiative guidelines was used to define renal function impairment [12]. Carotid ultrasonography was performed to measure CIMT and CAP. The institutional review board of Hanyang University Guri Hospital approved the study design and procedures.

### Carotid ultrasonography measurements

CIMT was measured from the lower arterial wall on longitudinal views of each distal common carotid artery during end-systole. The measurement of CIMT was achieved using automated software (Philips Healthcare, Andover, MA, USA). The average value of the right- and left-sided CIMTs was used for analysis. CAP was defined as the presence of an area with a ≥50% increase in the intima-media thickness compared to that of the neighboring vessel wall. CAP was sought and identified in both common carotid arteries on transverse views, and was measured on longitudinal views.

### Coronary and renal angiography

The standard approach for CAG in the hospital where this study was performed is femoral, and right radial approach is used in a minority of patients if the femoral approach is not available or the patients specially request the radial approach. The femoral artery approach was used for both the coronary and simultaneous renal angiography in this study. 5-Fr Judkins left and right diagnostic catheters (Cordis, Bridgewater, NJ, USA) were used for left and right coronary angiography, respectively. Renal angiography was performed using a 5-Fr Judkins right diagnostic catheter engaged in or directed to the renal artery ostium, with contrast medium flowing back from the renal artery. Both renal arteries were visualized in anterior-posterior projections. The degree of stenosis was measured using quantitative coronary angiography software (Siemens, Philadelphia, PA, USA). Significant CAD was defined as stenosis ≥70% in at least one coronary artery. Significant RAS was defined as stenosis ≥50% in at least one side.

### Statistical analysis

The subjects were divided into two groups according to the presence or absence of significant RAS. The student’s *t* test was used to compare continuous variables such as age, BMI, total cholesterol level and eGFR between the two groups. Variables with skewed distributions such as triglyceride level, high density lipoprotein (HDL) cholesterol level and CIMT were compared using Mann–Whitney *U* test. The *χ*^2^ test was used to compare binary variables such as hypertension, gender, diabetes, smoking, significant CAD, presence of CAP and CKD stage ≥3.

The model for predicting significant RAS was developed as following; the best-fit model predicting for significant RAS was determined using multiple logistic regression analysis, the model was validated to identify the degrees of optimism and variance, and finally, a scoring system for predicting significant RAS was developed using the coefficients from the regression formula of the best-fit model.

In order to find the best-fit model, we transformed the continuous variables into binary variables using the Youden index-J of the receiver operating characteristic (ROC) curve to maximize their discriminative powers of the best-fit model (e.g., age ≥67 years, BMI <22 kg/m^2^). Although dichotomization of continuous variables may cause bias and weaken the discriminative power of the model, we used this method because a model using binary or categorical variables would be more accessible than a model using continuous variables. The extent of CAP was defined as a 3-category variable (none, unilateral, bilateral). Then, multiple logistic regression analysis was performed with all binary variables available and the extent of CAP as a categorical variable, to reduce biases in variable selection. Backward variable selection using Wald statistics was performed to identify significant variables and reduce the best-fit model to acceptable events per variable [13]. The exclusion criterion for the backward selection process was set at *p* ≥ 0.10, to avoid excluding modestly significant variables.

Next, validation and calibration of the best-fit model were performed using bootstrapping methods. Bootstrap re-sampling is an effective technique for internal validation and calibration of a prediction model [14]. Validation using bootstrap re-sampling would estimate the likely performance of the model on a new sample of patients from a same clinical setting. Calibration would measure the degree of error between the predictive probability and observed probability of the model. The validation was performed with 1,000 re-samples drawn using the 0.632 bootstrap technique [15]. We programed the statistical software to calculate new optimal cut-off points for continuous variables, generate binary variables using the new cut-off values in each bootstrap sample and run a multiple logistic regression analysis using the backward selection method with all the new binary variables. Bootstrap models were built from each bootstrap sample. The estimate of optimism in the best-fit model was calculated from the following formula:

$$O=\frac{1}{M}\times \sum_{n=1}^{M}\left(C_{\mathrm{original}\;\mathrm{sample}}^{n}-C_{\mathrm{boostrap}\;\mathrm{sample}}^{n}\right)$$

where O is the estimate of optimism, C_original sample_ is the area under the curve (AUC) of the ROC curve of the bootstrap model in the original data, C_bootstrap sample_ is the AUC of the ROC curve of the bootstrap model in each bootstrap sample and M is the number of bootstrap samples.

Finally, using the best-fit model, we developed a risk scoring system for predicting significant RAS. Using the following formula for logistic regression analysis of the best-fit model:

$$\begin{array}{ll} \ln\frac{P}{\left(1-P\right)}= & \alpha +\beta_{1}X_{1}+\beta_{2}X_{2}+\beta_{3}X_{3}\ldots +\beta_{n}X_{n}\\ & \left(\mathrm{when}\;\mathrm{coefficient}\;\beta_{1}<\beta_{2}<\beta_{3}<\ldots <\beta_{n}\right) \end{array}$$

we assigned the integer closest to β_n_/β_1_ as the score for the variable X_n_. Then, we summated the integers to generate a risk score for predicting significant RAS in each patient, if variable X_n_ was present in the patient. ROC curve analysis was performed to evaluate the discriminative power and optimal cut-off point of the scores for predicting significant RAS. The goodness-of-fit of the risk scoring system for significant RAS was assessed using the Hosmer-Lemeshow test and Levenburg-Marquardt nonlinear regression analysis. The predicted probability of significant RAS was calculated from the following equation.

$$\mathrm{Probability}=\frac{1}{1+e^{-L}}$$

(when L = α + coefficient β × (risk score) in a logistic regression analysis)

All statistical analyses were performed with statistical software, R-3.0.1 for Windows. The rms package was used for logistic regression analysis and the ROCR, pROC and Epi packages were used to identify the optimal cut-off points for continuous variables and automatically transform the continuous variables into binary variables.

## Results

### Baseline characteristics of subjects

**Figure 1:**
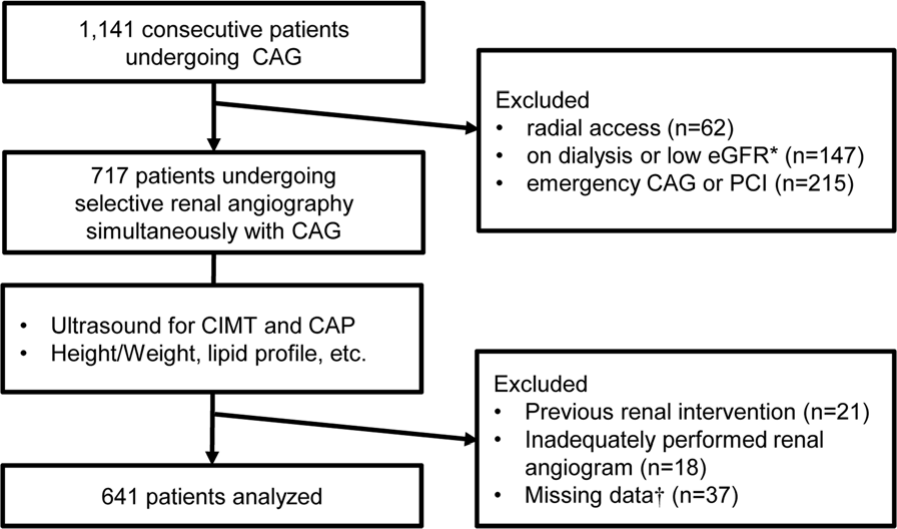
**Flowchart of the patients enrolled in the study.** *eGFR <15 ml/min/1.73 m^2^. † 8 cases without carotid ultrasonography results, 13 cases without data for height or body weight and 16 cases without laboratory data. CAG, coronary angiography; eGFR, estimated glomerular filtration rate calculated using the Modification of Diet in Renal Diseases equation; PCI, percutaneous coronary intervention; CIMT, carotid intima-media thickness; CA, carotid atherosclerotic plaque.

Of the 1141 patients who underwent CAG during the period of study, 641 patients remained for the final analysis (Figure 1). The mean age was 61.2 ± 12.5 years and 49% of the patients were male. Hypertension was present in 61.0% of patients, diabetes in 27.8%, and smoking in 27% of the patients. The number of anti-hypertensive medications (AHM) taken by a patient was 1.6 ± 1.0. The mean serum creatinine was 0.87 ± 0.37 mg/dl, and CKD stage ≥3 was present in 45 patients (7%). Total cholesterol level was 175.8 ± 41.1 mg/dl, HDL cholesterol level 47.3 ± 12.5 mg/dl and triglyceride level 148.6 ± 120.9 mg/dl. The mean CIMT was 0.89 ± 0.26 mm and CAP was present in 271 patients (42%). Significant CAD was present in 168 patients (26%) and significant RAS in 60 patients (9.4%). The baseline characteristics of patients with and without significant RAS are shown in Table 1. The mean age, number of AHM and CIMT were higher in patients with significant RAS than those without significant RAS, whereas BMI, total cholesterol level, HDL cholesterol level and eGFR were lower in patients with significant RAS than those without significant RAS. Triglyceride level was not different between the two groups. Hypertension, diabetes, CKD stage ≥3 and proteinuria were more prevalent in patients with significant RAS than those without significant RAS, whereas the proportions of males and current smokers were not different between the two groups. Significant CAD and the presence of CAP were more prevalent in patients with significant RAS than those without significant RAS.

**Table 1:** Baseline characteristics of patients undergoing coronary angiography

	**Without RAS ≥ 50% (n = 581)**	**With RAS ≥ 50% (n = 60)**	**P-value**
Age (years)	60.3 ± 12.4	70 ± 9.1	< 0.001
Male gender, n (%)	282 (48.5)	32 (53.3)	0.479
Hypertension, n (%)	348 (59.9)	43 (71.7)	0.049
Number of AHM	1.5 ± 1.1	2.2 ± 1.1	< 0.001
Diabetes, n (%)	152 (26.2)	26 (43.3)	0.005
Smoking, n (%)	155 (26.7)	18 (30)	0.581
BMI (kg/m^2^)	25.5 ± 3.4	24.1 ± 4.3	0.006
Total Cholesterol (mg/dl)	177.5 ± 40.9	159.7 ± 39	0.003
HDL Cholesterol (mg/dl)†	46.0 (39.0, 55.0)	42.5 (38.0, 51.5)	0.049
Triglyceride (mg/dl)†	122.0 (89.0, 173.0)	115.5 (81.3, 159.0)	0.343
eGFR (ml/min/1.73 m^2^)	111.5 ± 35.1	81.5 ± 34.7	< 0.001
CKD stage ≥3 n (%)	27 (4.6)	18 (30)	< 0.001
Proteinuria, n (%)	76 (13.1)	15 (25)	0.012
Significant CAD*, n (%)	124 (21.3)	44 (73.3)	< 0.001
CIMT (mm)†	0.84 (0.72, 0.98)	1.00 (0.85, 1.15)	< 0.001
CAP, n (%)	221 (38)	50 (83.3)	< 0.001

Results are shown as the mean ± SD or n (%).

The percentage inside the brackets indicates either the percentage in the group with RAS ≥50% or the percentage in the group without RAS ≥50%.

*CAD with stenosis ≥70% at least one coronary artery.

†Data for skewed variables are shown as the median (1st quartile, 3rd quartile).

RAS, renal artery stenosis; AHM, anti-hypertensive medications; BMI, body mass index; HDL, high-density lipoprotein; eGFR, estimated glomerular filtration rate; CKD, chronic kidney disease; CAD, coronary artery disease; CIMT, carotid intima-media thickness; CAP, the presence of carotid atherosclerotic plaque.

### Multiple logistic regression analysis for predictors of significant RAS

**Figure 2:**
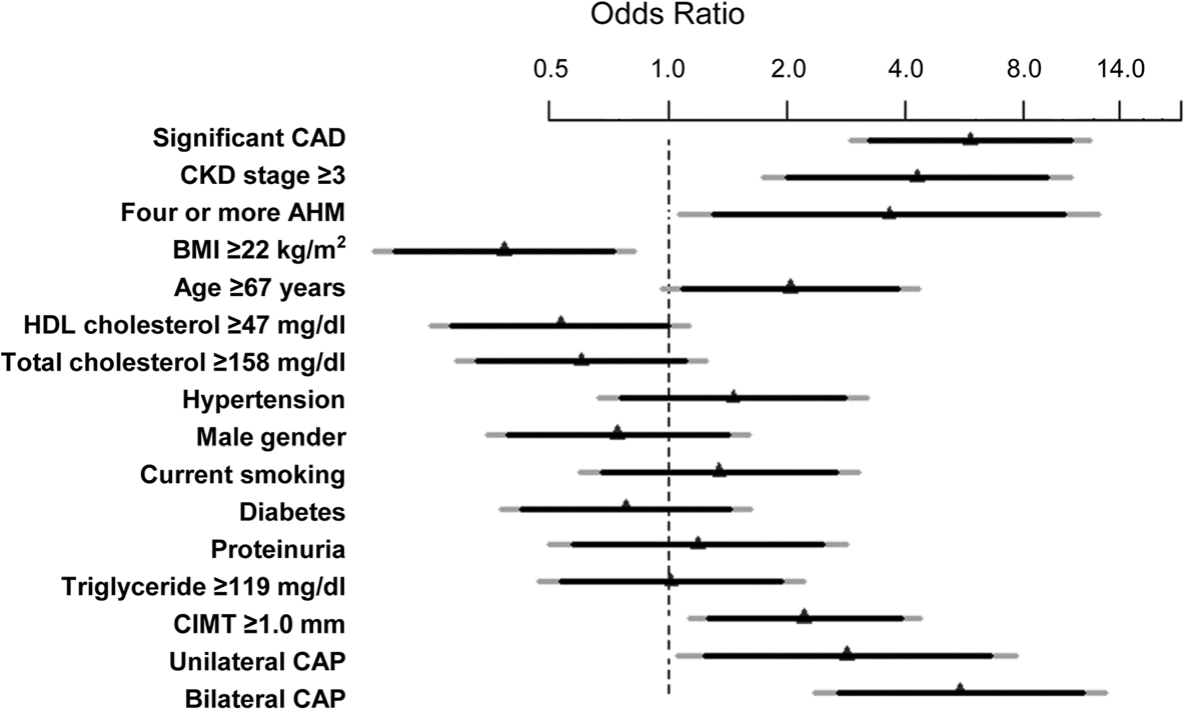
**Predictors of RAS ≥50%.** The odds ratio and CI are derived from multiple logistic regression analysis including all variables. The ruler is transformed into a log-scale. Triangles indicate OR, black bars 90% CI and grey bars 95% CI. Numbers inside the brackets indicate the optimal cut-off points for the continuous variables, derived from the Youden index-J of ROC curve analysis. Significant CAD, CKD stage ≥3, four or more AHM, CAP, CIMT ≥1.0 mm, BMI <22 kg/m^2^ and Age ≥67 years are significant predictor for RAS ≥50%. CAD, coronary artery disease; CKD, chronic kidney disease; AHM, anti-hypertensive medication; BMI, body mass index; HDL, high density lipoprotein; CIMT, carotid intima-media thickness; CAP, carotid atherosclerotic plaque; RAS, renal artery stenosis, CI, confidence interval.

The optimal cut-off values for continuous variables obtained by ROC curve analysis were CIMT 1.0 mm (AUC = 0.683, *p* = 0.002), age 67 years (AUC = 0.726, *p* <0.001), BMI 22 kg/m^2^ (AUC = 0.608, *p* = 0.003), total cholesterol level 158 mg/dl (AUC = 0.618, *p* = 0.002), HDL cholesterol level 47 mg/dl (AUC = 0.577, *p* = 0.128) and triglyceride level 119 mg/dl (AUC = 0.537, *p* = 0.323). Multiple logistic regression analysis including with all the binary and categorical variables identified significant CAD (odds ratio (OR) 5.8, *p* <0.001), CKD stage ≥3 (OR 4.3, *p* = 0.002), four or more AHM (OR 3.6, *p* = 0.039), BMI ≥22 kg/m^2^ (OR 2.6, *p* = 0.014), CIMT ≥1.0 mm (OR 2.2, *p* = 0.020), unilateral CAP (OR 2.9, *p* = 0.039) and bilateral CAP (OR 5.5, *p* <0.001) as significant predictors of significant RAS (Figure 2). Among the significant predictors, significant CAD, CKD stage ≥3, four or more AHM and bilateral CAP were stronger predictors of significant RAS. Age ≥67 years and HDL cholesterol level ≥47 mg/dl were marginally significant predictors of significant RAS. Total cholesterol level ≥158 mg/dl, hypertension, male gender, current smoking, diabetes, proteinuria and triglyceride level ≥119 mg/dl were not significant predictors of significant RAS. The average variable inflation factor of all the variables included in the multiple logistic regression analysis was 1.19 and no variable inflation factor of any variable exceeded 1.4. In multiple logistic regression analysis with backward selection, significant CAD, unilateral or bilateral CAP, CKD stage ≥3, four or more AHM, CIMT ≥1.0 mm, age ≥67 years and BMI < 22 kg/m^2^ remained as predictors of significant RAS (Table 2).

**Table 2:** Multiple logistic regression analysis for independent predictors of RAS ≥50%

**Predictors**		**Coefficient β**	**OR (95% CI)**	**p-value**
Significant CAD*		1.724	5.6 (2.9-11.0)	<0.0001
Extent of CAP	One side	0.958	2.6 (1.0-6.8)	0.0503
	Both sides	1.584	4.9 (2.1-11.1)	0.0002
CKD Stage ≥3		1.566	4.8 (2.1-11.0)	0.0002
Four or more AHM		1.563	4.8 (1.5-14.8)	0.0069
CIMT ≥1.0 mm		0.849	2.3 (1.2-4.5)	0.0109
Age ≥ 67 years		0.831	2.3 (1.2-4.6)	0.0173
BMI < 22 kg/m^2^		0.872	2.4 (1.2-4.9)	0.0174

Multiple logistic regression analysis was performed using a backward selection method.

*Stenosis ≥70% in at least one coronary artery.

OR, odds ratio; CI, confidence interval; CAD, coronary artery disease; AHM, antihypertensive medications; eGFR, estimated glomerular filtration rate; CIMT, carotid intima-media thickness; CAP, carotid atherosclerotic plaque.

### Scoring system for significant RAS

**Figure 3:**
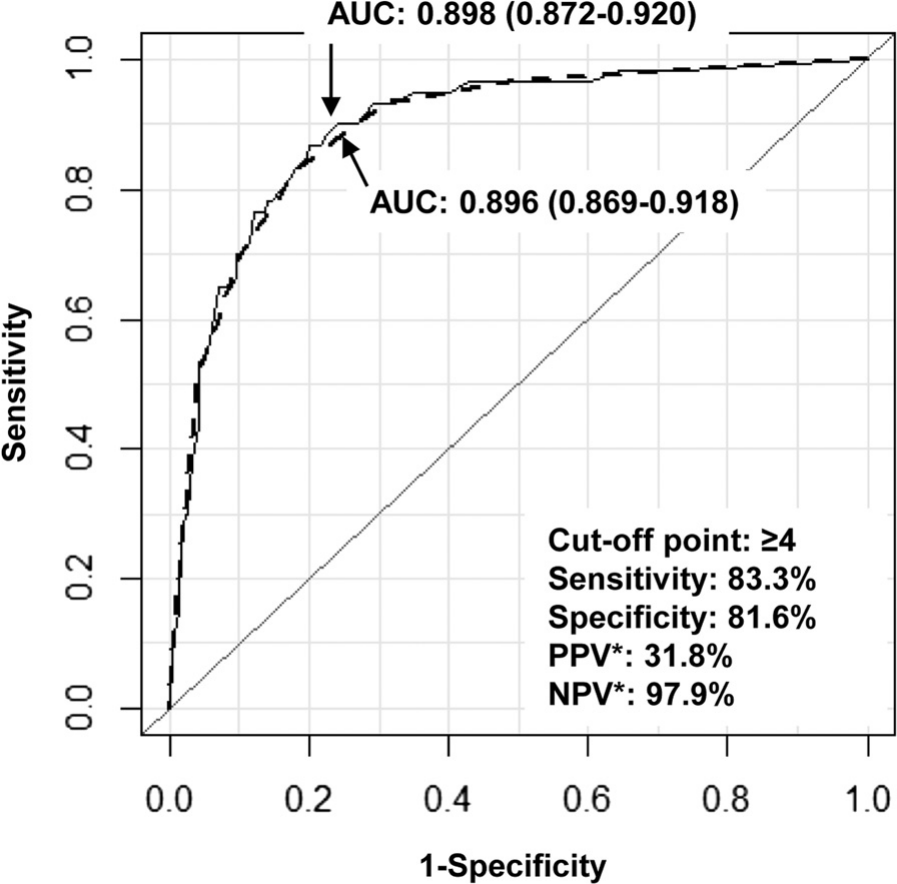
**Performance of the scoring system for predicting RAS ≥50%.** The broken line indicates the ROC curve of the scoring system and the unbroken line indicates the ROC curve of the best-fit model. The difference between the two AUCs is 0.002, and is not significant (*p* = 0.69, DeLong method). The numbers inside the brackets indicate the 95% confidence intervals of the AUCs. *The PPV and NPV are estimated using a 9.4% prevalence of RAS ≥50%. AUC, area under curve of ROC curve; ROC, receiver operating characteristics; PPV, positive predictive value; NPV, negative predictive value.

Using the results of the multiple logistic regression analysis with backward selection, we developed a scoring system for predicting significant RAS (Table 3). To produce the scoring system, we assigned the simplest integers proportional to the coefficient β of each predictor. The smallest coefficient β was 0.831 for age ≥67 years and the largest one was 1.724 for significant CAD. The ratios of the coefficients of predictors to the smallest coefficient were therefore all between 1 and 2. We assigned a score of 1 to unilateral CAP, CIMT ≥1.0 mm, age ≥67 years and BMI <22 kg/m^2^, and a score of 2 to significant CAD, bilateral CAP, CKD stage ≥3 and four or more AHM. The total scores ranged from 0 to 11. In ROC curve analysis, the scoring system for significant RAS showed an AUC of 0.896 (95% confidence interval 0.869 - 0.918), which was not significantly different from the AUC of the best-fit model (AUC = 0.898, difference = 0.002, *p* = 0.69 using the DeLong method). The scoring system showed sensitivity of 83.3% and specificity of 81.6% at a cut-off point of ≥4 Using the same cut-off point and a prevalence of significant RAS 9.4%, the positive predictive value was 31.8%, and the negative predictive value was 97.7% (Figure 3). The predicted frequency of significant RAS using the scoring system agreed well with the observed frequency of significant RAS. The Hosmer-Lemeshow test for the goodness-of-fit of the scoring system showed *p* = 0.881, and the coefficient of determination between the predicted frequency and observed frequency using Levenburg-Marquardt non-linear regression analysis was *R*^*2*^ = 0.957 (Table 4).

**Table 3:** Scoring system for predicting RAS ≥50%

**Predictor**	**Criteria**	Score*
CAD	No stenosis ≥70% on coronary arteries	0
	Stenosis ≥70% on at least one coronary artery	2
CKD	Stage <3	0
	Stage ≥3	2
AHM	Less than 4	0
	4 or more	2
BMI	≥22 kg/m^2^	0
	<22 kg/m^2^	1
Age	<67 years old	0
	≥67 years old	1
CAP	None	0
	Present at one side	1
	Present at both sides	2
CIMT	<1.0 mm	0
	≥1.0 mm	1

This scoring system should be independently validated, before used in clinical practice.

*Total scores range from 0 to 11.

CAD, coronary artery disease; CKD, chronic kidney disease; AHM, anti-hypertensive medication; BMI, body mass index; CIMT, carotid intima-media thickness; CAP, carotid atherosclerotic plaque.

**Table 4:** Observed and predicted frequencies of RAS ≥ 50% using the scoring system

		**Without RAS ≥50%**		**With RAS ≥50%**	
**Score**	**Numbers of patients (n)**	**Observed frequency (n)**	**Predicted frequency (n)**	**Observed frequency (n)**	Predicted frequency* (n)
0	156	155	155.12	1	0.88
1	145	144	144.17	1	1.83
2	106	104	103.01	2	2.99
3	77	71	72.26	6	4.74
4	56	48	48.75	8	7.24
5	45	34	33.68	11	11.32
6	28	14	15.90	14	12.10
7	21	9	7.71	12	13.29
8	7	2	1.43	5	5.57
9	0	0	0	0	0
10	0	0	0	0	0
11	0	0	0	0	0

*(Number of patients) × 1/[1 + Exp(-L)], when L = -5.175 + 0.817 × (score).

*p* = 0.881, Hosmer-Lemeshow test.

Coefficient of determination *R*^2^ = 0.957 for agreement with the observed frequency, Levenburg-Marquardt non-linear regression method.

### Validation and calibration of the best-fit model

**Figure 4:**
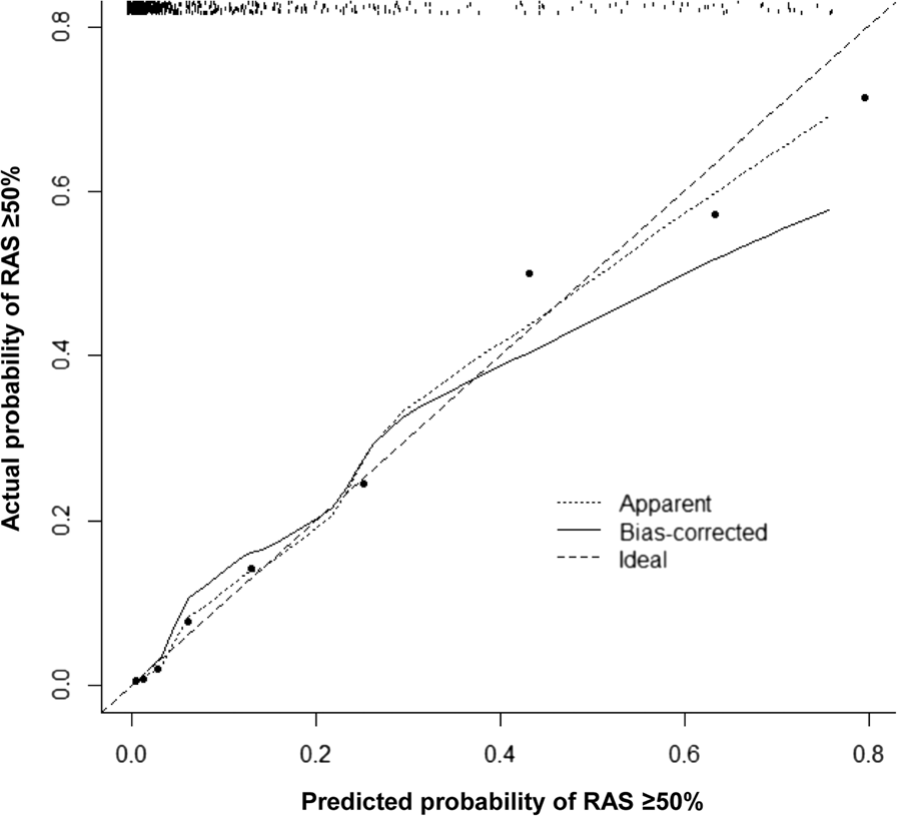
**Calibration plot for the model for predicting RAS ≥50%.** The plot illustrates the accuracy of the best-fit model (“Apparent”) and the bootstrap model (“Bias-corrected”) for predicting RAS ≥50%. Locally weighted scatterplot smoothing was used to illustrate the relationships of the two models with the ideal line. Both plots are slightly non-linear and agree well in low predicted probabilities of RAS ≥50%, but the disagreement between the two plots grows with the predicted probability of RAS ≥50%. The 0.9 quantile absolute error of the predicted probability is 0.047. The black dots illustrate the relationship between the predicted probability and observed probability of the scoring system for predicting RAS ≥50% in the original data set. The *r*^2^ of linear regression of the dots is 0.982.

The apparent AUC of the best-fit model derived from multiple logistic regression analysis with backward selection was 0.898 (95% confidence interval 0.872-0.918). Validation of the best-fit model with 1000 bootstrap re-samples showed that the average optimism was 0.023 and the adjusted AUC of the best-fit model was 0.875. The calibration plots of the best-fit model and the bootstrap model are shown in Figure 4. The estimates of both models were slightly non-linear, with the bootstrap model being slightly more non-linear than the best-fit model. Both models agreed well with the ideal line when the predicted probability of significant RAS was low, but the disagreement of the bootstrap model increased with increasing the predicted probability of significant RAS. However, the mean absolute error and 0.9 quantile absolute error of the predicted probability were 0.028 and 0.047 respectively, suggesting only a small degree of bias from overfitting in the best-fit model.

## Discussion

### Main findings

We found that the extents of CAP and CIMT were independent predictors of significant RAS in patients undergoing CAG. The prevalence of significant RAS increased with the presence of CIMT and the extent of CAP. The model for predicting significant RAS which included significant CAD, CKD stage ≥3, four or more AHM, age ≥67 years, BMI <22 kg/m2, CIMT ≥1.0 mm, and the extent of CAP showed a high discrimination power and a small degree of bias. The predictive frequency of the scoring system agreed well with the observed frequency of significant RAS.

### Prevalence and predictors of significant RAS

The prevalence of significant RAS was 9.4%, which is similar to the findings of previous studies [3,5,9,11,16,17]. However, the actual prevalence of significant RAS may be higher, because we excluded patients undergoing emergency PCI and those with a history of renal artery intervention.

Hypertension and diabetes, classic risk factors for atherosclerosis, were not strongly associated with significant RAS in this study because they were already reflected by other predictors, namely significant CAD, CIMT, extent of CAP, old age, four or more AHM. The high prevalence of hypertension among patients undergoing CAG may also have contributed to the weak association between significant RAS and hypertension. Instead, four or more AHM indicating severe, uncontrolled hypertension was a strong predictor of significant RAS. The BMI and total cholesterol levels were paradoxically lower in patients with significant RAS than those without significant RAS. This may be because the BMI and total cholesterol level also partially reflect muscle mass and nutritional status [18,19]. Patients with significant RAS often have multiple co-morbidities and poor general health. Prezewlocki et al. [10] also reported that BMI <25 kg/m^2^ was a predictor of significant RAS.

Carotid ultrasonography parameters, such as CAP and CIMT, are known as the predictors of cardiovascular disease [7,8,20], and the relationship between RAS and systemic atherosclerosis is well established [16]. The association between CIMT and RAS, however, has only been reported in a few studies [21,22], and the value of the extent of CAP for predicting RAS has not been reported until now. This is the first study using a multiple logistic regression model to report CIMT and the extent of CAP measured by carotid ultrasonography as independent predictors of significant RAS in patients undergoing CAG.

### Model for predicting RAS

RAS contributes to severe but correctable, hypertension and to left ventricular hypertrophy. Although RAS is an independent risk factor for cardiovascular mortality, screening for RAS in asymptomatic patients is currently controversial, because none of the large randomized trials to date have shown clinical benefits associated with renal artery stenting compared with medical therapy in patients with RAS [23,24]. Routine “drive-by” renal angiography in all patients undergoing elective CAG is especially difficult to support because of the lack of evidence of benefit and the low prevalence of RAS. A distinction should be made, however, between identifying patients with significant RAS and selecting patients for renal artery intervention. It is important to identify patients with significant RAS who are at increased risk of cardiovascular events and require close observation [25]. RAS is an independent predictor of mortality in patients with cardiovascular disease [1,2,25]. From this point of view, it may be useful to determine indications for performing renal angiography at the time of CAG in selected patients undergoing CAG.

The American Heart Association advises renal angiography at the time of CAG in patient with multi-vessel CAD or PAD [6]. The reported prevalence of significant RAS ranges from 10% to 36% in patients with triple-vessel CAD [3,9-11,22] and from 21% to 55% in patients with PAD [9,11,25]. Models for predicting significant RAS that include multiple clinical predictors may enable more accurate selection of patients for renal angiography. Several studies have proposed prediction models for significant RAS in patients undergoing CAG [9-11]. Clinical predictors including age, hypertension, BMI, number of AHM, CAD, PAD, serum creatinine level and eGFR have been used in these models. However, these prediction models were either too complicated or did not provide a sufficiently predictive performance to be applied to clinical practice.

Duplex ultrasonography is a safe and acute non-invasive screening tool for RAS. The sensitivity and specificity of duplex ultrasonography were already 92.5% and 95.7%, respectively, in a 1997 study of patients suspected to have RAS [26]. Duplex ultrasonography will be a superior screening modality to any clinical predictors or scoring methods, if a patient is suspected to have RAS. Nevertheless, a scoring system for predicting RAS can still be a useful tool for clinicians to estimate the risk of RAS in asymptomatic patients undergoing CAG.

Carotid ultrasonography is a simple, non-invasive tool frequently used in current clinical practice to evaluate cardiovascular risk [27]. Our scoring system for predicting significant RAS included CIMT and CAP measured by carotid ultrasonography, and showed better sensitivity and specificity than scoring systems in the previous studies. The high negative predictive value and moderate positive predictive value may enable use of the scoring system as a quick decision-making tool for a physician to undertake a definite diagnostic testing for significant RAS. The goodness-of-fit between the predicted and observed frequency of significant RAS was high in our model. The scoring system was also made as simple as possible, so that it could easily be applied to clinical practice. All the items of the scoring system were assigned in the simplest integers, 1 or 2, and the score range was 0 to11.

We also validated our model with bootstrap re-sampling technique. Validation and calibration procedures are required for a prediction model to be useful in clinical practice. Przewlocki et al. [10] performed validation of their model by random splitting of the data. However, other previous studies that reported models for predicting RAS did not perform validation procedures. Bootstrap re-sampling is a more effective technique for validating a prediction model than data splitting [14], and the 0.632 bootstrap technique used in our validation procedures is a variant bootstrap method that can provide very similar validation to that obtained using an independent data set [26]. A small degree of optimism was observed, and the disagreement in validation and calibration between the predicted probability and actual probability increased with increasing predicted probability. However, the bias from overfitting was acceptable in this study. We believe that our prediction model can be a useful tool for evaluating the risk of significant RAS in patients undergoing CAG.

### Limitations

Our study was performed in a single center and may therefore contain referral bias. Although the patients were included consecutively in the study, the exclusion of patients because of radial approach, emergency PCI, end-stage renal disease, previous renal artery intervention, or inadequately performed renal angiography may have affected the recorded prevalence of significant RAS. Carotid ultrasonography is a simple and non-invasive tool for assessing the extent of atherosclerotic diseases, but, is still not performed routinely in patients undergoing CAG. Although the scoring system can be used for pre-procedural estimation of the probability of RAS, the indications for renal artery intervention in asymptomatic patients still need to be established for the prediction model to be relevant to improving clinical outcomes. Finally, although we validated our model, internally with bootstrap re-sampling technique, the proposed scoring system should be externally validated before being used in routine clinical practice.

## Conclusions

CIMT and CAP measured by carotid ultrasonography are independent predictors of significant RAS in patients undergoing CAG. The proposed model for predicting significant RAS which includes the carotid ultrasonography parameters and other independent predictors showed good diagnostic performance and only a small amount of bias, and may be a useful tool for deciding whether a definite diagnostic procedure is needed at the time of CAG. Further investigation is needed for independent validation of our model.

## Abbreviations

RAS: Renal artery stenosis; CAD: Coronary artery disease; CAG: Coronary angiography; CIMT: Carotid intima-media thickness; CAP: Carotid atherosclerotic plaque; PAD: Peripheral artery disease; PCI: Percutaneous coronary intervention; BMI: Body mass index; eGFR: Estimated glomerular filtration rate; CKD: Chronic kidney disease; HDL: High-density lipoprotein; ROC: Receiver operating characteristics; AHM: Antihypertensive medications; OR: Odds ratio; AUC: Area under the curve.

## Competing interests

The authors declare that they have no competing interests.

## Authors’ contributions

YL conceived the basic idea, designed the study, and wrote the manuscript. JHS and HCP helped select the patients, performed angiography and carotid ultrasonography, and analyzed the data. SGK helped perform the statistical analysis and drafted the manuscript. SIC helped select the patients, performed angiography and directed the whole process of the study. All authors read and approved the final manuscript.

## Pre-publication history

The pre-publication history for this paper can be accessed here:

http://www.biomedcentral.com/1471-2369/15/60/prepub
